# Retrospective study on the trajectories of lower limb volume after outpatient-based complex decongestive therapy in post-operative gynecological cancer patients with lymphedema

**DOI:** 10.1007/s00520-023-07783-7

**Published:** 2023-05-06

**Authors:** Ayano Masui, Tsuyoshi Harada, Yoshihiro Noda, Ryo Soeda, Hisashi Kida, Tetsuya Tsuji

**Affiliations:** 1 Department of Rehabilitation Medicine, Keio University Graduate School of Medicine, Shinjuku, Tokyo, Japan; 2 Department of Rehabilitation Medicine, National Cancer Center Hospital East, Kashiwa, Chiba Japan; 3 Department of Neuropsychiatry, Keio University School of Medicine, Shinjuku, Tokyo, Japan; 4 Department of Rehabilitation, Tsurumaki-Onsen Hospital, Hadano, Kanagawa Japan; 5 Department of Rehabilitation Medicine, Keio University School of Medicine, 35 Shinanomachi, Shinjuku-ku, Tokyo, 160-8582 Japan; 6 Lymphedema Treatment Center at, Keio University Hospital, Shinjuku, Tokyo, Japan

**Keywords:** Rehabilitation, Group-based trajectory modeling, Cancer survivors, Outpatient

## Abstract

**Purpose:**

To determine the effect of outpatient-based complex decongestive therapy in patients with secondary lower limb lymphedema (LLL) after gynecologic cancer surgery using group-based trajectory modeling (GBTM), and to examine factors predictive of the treatment course.

**Methods:**

This retrospective study included participants who underwent surgery for gynecological cancer with pelvic lymph node dissection and subsequently visited the outpatient clinic for the treatment of stage II LLL according to the International Society of Lymphology. The improvement rate of edema at the initial visit and 3, 6, and 12 months later was assessed by calculating the volume of the lower extremity using the circumferential method. For evaluation of the patterns of treatment course, logistic regression analysis was performed after group estimation by the trend of the treatment course using GBTM.

**Results:**

A total of 148 women (mean age 60.6 years (standard deviation: 13.4 years)) were analyzed. Three improvement trajectories were identified: (1) no response group, with worsening rather than improvement (*n* = 26); (2) moderate response group, with a slow improvement rate (*n* = 89); and (3) high response group, with a high improvement rate (*n* = 33). In addition, adherence to compression therapy at 3 months post-intervention was found to be a predictor in the no response group.

**Conclusions:**

GBTM estimated that there are three patterns of the treatment course in patients with LLL after gynecologic cancer surgery. Adherence to compression therapy at 3 months post-intervention is a predictor of the treatment effectiveness.

## Introduction

Lymphedema results from an inability to process microvascular filtrate due to dysfunction of the lymphatic system and abnormal lymphatic transport capacity [1]. It is broadly classified into primary and secondary forms [2]. In Japan, most cases are secondary lymphedema that occurs as a sequela of cancer treatment [3]. Although there is no unified view on the incidence of lymphedema due to the lack of standardized diagnostic and evaluation criteria, previous studies conducted in Japan have reported an incidence of 15.2–30.2% for secondary lower limb lymphedema (LLL) [4, 5]. LLL has various adverse effects, including physical effects such as pain and heaviness associated with swelling of the lower extremities, sensation of heat and redness, as well as limitation of activity [6–8], and psychological effects such as anxiety and insomnia [6, 9]. Additionally, cancer survivors with lymphedema need to purchase elastic garments and bandages for their treatment. Hence, the average cost of medical care tends to be high, indicating a significant financial burden [10, 11]. The results of previous studies have indicated that early detection and treatment of lymphedema not only reduces medical costs but also improves the quality of life of patients [11, 12]. Therefore, we consider that clarification of the patterns of the response to treatment will facilitate early detection and treatment of patients with a poor therapeutic response and will arrest the progression of lymphedema and reduce the economic burden on the patients.

Globally, complex decongestive therapy (CDT) is recommended as the standard conservative therapy for LLL [1]. CDT consists of skin care, manual lymphatic drainage, compression therapy, and exercise therapy under compression. CDT is performed in two phases: an intensive treatment phase with intensive intervention under professional supervision and a maintenance phase, in which the condition is maintained primarily through patient self-management. CDT can also be provided on an outpatient basis if deemed appropriate by the medical team, depending on the condition of the patient's affected limb and the patient’s needs [1, 13]. A previous study reported that intensive treatment results in decrease of the LLL within 24 months [14]. However, in that study, the standard deviation (SD) of the percentage volume loss of the affected limbs at each assessment time point was large, ranging from ± 17.2 to 28.6%. In other words, the percentage of excess volume and rate of volume loss of the affected extremities were aggregated to an average value. Hence, potential progress patterns with treatment might have been overlooked. Therefore, clarification of more detailed patterns of treatment courses would be useful for considering tailor-made treatment plans for each patient.

One statistical method that has recently received attention is group-based trajectory modeling (GBTM), developed by Nagin et al. [15]. GBTM aims to identify clusters of individuals with similar trajectories that cannot be identified a priori. Recently, GBTM analysis has been used in medical research to facilitate causal inference in epidemiological observational studies where randomization of treatment conditions is impossible and to capture heterogeneity in treatment responses to interventions [16, 17]. This study is aimed at analyzing the effect of outpatient-based complex decongestive therapy in patients with secondary LLL after gynecologic cancer surgery using GBTM, to identify patterns of the treatment course, and examining predictors of treatment effect.

## Methods

### Design and participants

This retrospective study included patients with secondary LLL after gynecological cancer surgery who were attending or had attended the lymphedema outpatient clinic or cancer rehabilitation outpatient clinic of the Tumor Center at Keio University Hospital between January 2012 and August 2020. The eligibility criteria were as follows: (1) patients with secondary LLL of International Society of Lymphology (ISL) lymphedema stage II following pelvic lymph node dissection, (2) no prior treatment for LLL within the past year retroactively from the time of the initial visit, (3) those who we were followed for at least 3 months, and (4) aged 20 years or older. The exclusion criteria were (1) patients who had difficulty in understanding or communicating, or had cellulitis, deep vein thrombosis, arteriosclerosis obliterans, renal disease, or cardiac disease at the initial visit; (2) patients who developed recurrent or multiple cancers during the observation period; and (3) patients who requested to withdraw from the study. This study was approved by the Ethics Review Committee of Keio University School of Medicine (20200009) and was performed in accordance with the Declaration of Helsinki. An opt-out method was used for obtaining consent for study participation because of its retrospective nature.

### Treatment for LLL

In this study, the intensive and maintenance phases were conducted as outpatient visits. In the intensive phase, the lymphedema team treated the patients for approximately 3 months after the initial visit, followed by a subsequent maintenance phase, which included self-care and care by physicians. The team consisted of rehabilitation physicians, physical therapists, and nurses who had attended the prescribed lymphedema training and received certification as required by public medical insurance. In the intensive phase, the duration of each intervention was 40–60 min, and the frequency of intervention was 1–3 times per month depending on the severity of the edema. The interventions included, first, an explanation of the condition to the patient by the rehabilitation physician; instruction on skin care methods and daily living using pamphlets; instructions on multilayer bandaging, selection, and application of elastic garments; instructions on exercising while using the bandages; and instruction on self-lymphatic drainage, as needed. Compression therapy was performed according to the initial management of lymphedema described in Lymphoedema Framework Best Practice for the Management of Lymphoedema [13]. During the maintenance phase, rehabilitation physicians provided medical care once every 3–6 months, with each visit lasting 20–30 min, depending on the patient’s progress. The evaluation included skin care and compression therapy adherence; confirmation of the application of multi-layer bandages and elastic garments; an interview to determine the status of exercise under compression, weight measurement, and measurement of the circumference of the lower extremity using a tape measure; and evaluation of edema and skin hardness by palpation.

### Measurements

#### Improvement rate of lower limb volume (IRLV) (at the initial visit and at 3, 6, and 12 months after intervention)

Lower limb volume was calculated using the truncated cone formula with circumferential measurements of the thigh and lower leg [18, 19], and the total of the two volumes in the same leg was defined as the lower limb volume. Lower limb circumference was measured at a total of four locations: 20 cm and 10 cm above the patella for the femoral circumference and 10 cm and 20 cm below the patella for the lower leg circumference. IRLV was calculated as follows using the volume at each evaluation time point.

$$\mathrm{IRLV}=\left(\mathrm{Initial}\;\mathrm{volume}-\mathrm{Volumes}\;\mathrm{after}\;3,\;6\;\mathrm{and}\;12\;\mathrm{months}\;\mathrm{of}\;\mathrm{intervention}\right)\div\mathrm{Initial}\;\mathrm{volume}\times100(\%)$$

#### Patient characteristics

Age, weight, diagnosis, medical history (presence or absence of orthopedic disease, presence or absence of cellulitis), presence or absence of chemotherapy (preoperative or postoperative), and presence or absence of radiation therapy (preoperative or postoperative) were investigated using the patients’ medical records. ISL stage, the affected side at the first visit, the number of days from surgery to the first visit to the lymphedema outpatient clinic, the time from lymphedema onset to the first outpatient visit, the number of lymphedema team interventions (number of physician visits + number of physical therapy interventions), lower extremity edema grade and skin hardness grade at the first visit and at 3, 6, and 12 months, and compression therapy adherence at 3 months after intervention (adherence) were studied and evaluated from the patients’ medical records. Adherence was rated on a two-point scale of good and poor, based on patients’ self-reported average compression therapy status over 3 months from the initial visit, using the methods of previous studies [20, 21]. Adherence was considered good if the patient performed some form of compression therapy both during the day and at night. If the patient performed some form of compression therapy during either only the day or only at night, or if the patient did not perform any form of compression therapy during both the day and at night, the patient was classified as having poor adherence.

### Statistical analyses

Descriptive statistics are presented as the number of people and mean ± standard deviation. Regarding selection of the best model for IRLV, using the GBTM analysis [15, 22, 23], the optimal model in this study was selected using the following procedure. In GBTM analysis, data containing defects can be analyzed only when the missing data is missing completely at random (MCAR) [15]. Little’s MCAR test (MCAR test) was first performed to confirm whether or not the missing data in this study were MCAR [24]. The optimal number of groups (number of trajectories) in the model was selected based on the Bayesian information criterion (BIC) and the logged Bayes factor (2ΔBIC) values. The shape (polynomial degree) of each trajectory in the group with the largest BIC was then specified. The final model selection was made using the criterion of average posterior probability (AvePP) greater than 0.7 for all trajectories. To examine the factors associated with each trajectory of IRLV, a logistic regression analysis was conducted using the trajectories estimated as categorical variables by GBTM analysis as the objective variables, and the endpoints with *p* values less than 0.15 in univariate analysis (the high response group vs. moderate response group, and the no response group vs. moderate response group) were used as explanatory variables in logistic regression analysis. In order to extract explanatory variables according to sample size, univariate analysis was performed according to the scale of the variables. Additionally, each variable was entered into a logistic regression analysis, noting multicollinearity among the variables. The statistical significance level was set at two-tailed 5%. JMP® 15 (SAS Institute Inc., Cary, NC, USA) was used for descriptive statistics and logistic regression analysis. R version 3.6.3 for the MCAR test and SAS 9.4 (SAS Institute Inc.) were used for the GBTM analyses.

## Results

### Participant characteristics

Among 299 patients who were initially included, 205 met the eligibility criteria. Excluding the 57 cases that met the exclusion criteria, the final analysis included 148 cases (including 48 cases with partially missing data). Basic information, medical information, and information on lymphedema treatment for the analyzed cases are shown in Table 1. Regarding adherence, 110 (76.4%) of the patients had good adherence, 34 (23.6%) had poor adherence, and data for 4 were incomplete.

**Table 1:** Patient characteristics (*N* = 148)

Age (years) mean ± SD			60.6 ± 13.4
Weight at initial visit (kg) mean ± SD			57.0 ± 11.9
Diagnostic name (%)		Endometrial cancer	58 (39.2)
		Cervical cancer	59 (39.9)
		Ovarian cancer	25 (16.9)
		Uterine sarcoma	1 (0.7)
		Endometrial + cervical cancer	2 (1.4)
		Endometrial + ovarian cancer	3 (2.0)
Medical history (%)		Orthopedic disorders	26 (17.6)
		Cellulitis	32 (21.6)
Adjuvant therapy (%)		Preoperative chemotherapy	2 (1.4)
		Postoperative chemotherapy	71 (48.0)
		Preoperative radiotherapy	0 (0.0)
		Postoperative radiotherapy	21 (14.2)
ISL stage (%)	II	Early stage	90 (60.8)
		Late stage	58 (39.2)
Site of lymphedema		Left	43 (29.0)
		Right	47 (31.8)
		Both sides	58 (39.2)
Median days between surgery and initial visit (range)			1747.5 (43–16571)
Time from edema onset to initial visit (%)		Within 6 months	82 (55.4)
		Less than 1 year	13 (8.8)
		Less than 2 years	23 (15.5)
		Over 2 years	23 (15.5)
		Uncertain	7 (4.7)

Abbreviations: *ISL*, International Society of Lymphology; *SD*, standard deviation. Descriptive statistics are presented as the number of people (%), mean ± SD, and median (range)

### Optimal model selection of IRLV by GBTM analysis

**Fig. 1:**
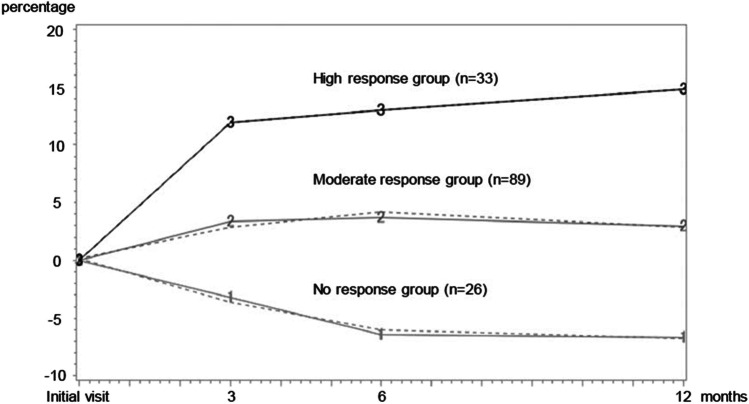
Trajectories of the three progress patterns with treatment, estimated by group-based trajectory modeling (GBTM). The solid line in the figure is the average improvement rate of lower limb volume (IRLV) for subjects in each group, and the dotted line is the estimated value by GBTM. The group with the lower most trajectory in the figure is the no response group (*n* = 26), which showed no improvement and instead worsened during the intervention, accounting for 19.2% of all subjects. The group with the middle trajectory in the figure is the moderate response group (*n* = 89), which showed gradual improvement and included 57.6% of all subjects. The group with the highest trajectory in the figure is the high response group (*n* = 33), which had a high improvement rate and continued to improve up to 12 months later, and included 23.2% of all subjects

The significant difference in the MCAR test was *p* = 0.11, and the null hypothesis that the missing data were MCAR was not rejected. In other words, since the missing data in this study were MCAR, GBTM analysis was performed. The results and rationale for the optimal model selection based on GBTM analysis are presented in Table 2. The optimal model in this study included three groups: (1) no response group, with worsening rather than improvement (*n* = 26); (2) moderate response group, with a slow improvement rate (*n* = 89); and (3) high response group, with a high improvement rate (*n* = 33) that met the criteria. The trajectories for each of the three groups estimated from GBTM analysis are shown in Fig. 1, and the mean IRLV for each group at each evaluation time point is shown in Table 3.

**Table 2:** Rationale for optimal model selection in group-based trajectory modeling

Number of trajectories	BIC	2ΔBIC	AvePP
1	-1735.4		
2	-1657.8	155.2	All groups > 0.7
**3**	**-1613.4**	**88.8**	**All groups > 0.7**

Abbreviations: *AvePP*, average posterior probability; *BIC*, Bayesian information criterion; *2ΔBIC*, the logged Bayes factor; Values in bold indicate optimal number of trajectories and rationale

**Table 3:** Mean improvement rate (%) in lower limb volume per group at each evaluation time point

Group	Initial visit	3 months	6 months	12 months
High response	-	11.8 (10.3 to 13.4)	13.0 (11.4 to 14.6)	14.7 (13.0 to 16.5)
Moderate response	-	3.3 (2.0 to 3.6)	3.7 (3.1 to 5.2)	2.9 (1.5 to 4.1)
No response	-	-3.2 (-4.9 to -2.3)	-6.3 (-7.8 to -4.1)	-6.6 (-9.0 to -4.4)

Descriptive statistics are presented as the mean percentage (%) and 95% confidence intervals

### Factors associated with each trajectory of IRLV

The predictors associated with each trajectory were examined. The results of the univariate analysis conducted to select variables to be entered into the logistic regression analysis are presented below. In the high response group (vs. moderate response group), ISL stage (*p* = 0.03) and history of cellulitis (*p* = 0.09) were extracted as variables, while in the no response group (vs. moderate response group), diagnostic name (*p* = 0.14) and adherence (*p* = 0.001) were extracted as variables (*p* < 0.15). The results of the logistic regression analysis are shown in Table 4. As a result, adherence (odds ratio 3.76, 95% confidence interval 1.41–10.05, *p* = 0.008) was extracted as an independent factor associated with the no response group. On the other hand, no significant factors were extracted in relation to the high response group.

**Table 4:** Logistic regression analysis for predictors of groups with a high response (a) and no response (b)

Variables	OR	95% CI	*p*-value
a. Predictors of high response			
ISL late stage (vs. early)	2.23	0.97 to 5.10	0.056
Cellulitis (vs. without cellulitis)	0.42	0.13 to 1.34	0.14
b. Predictors of no response			
Diagnostic name			
Cervical (vs. endometrial)	1.70	0.53 to 5.37	0.36
(vs. ovarian/others)	0.70	0.22 to 2.22	0.55
Endometrial (vs. ovarian/others)	0.41	0.11 to 1.46	0.17
**Poor adherence (vs. others)**	**3.76**	**1.41 to 10.05**	0.008^*^

Abbreviations: *CI*, confidence interval; *OR*, odds ratio; *ISL*, International Society of Lymphology; ^*^: *p* value < 0.05; Values in bold indicate adherence was extracted as an independent factor associated with the no response group

## Discussion

In this study, GBTM analysis estimated three trajectories as being the best model for IRLV. Additionally, adherence was extracted as a factor associated with the no response group. No previous studies examining treatment course patterns in patients with secondary LLL have been found, and this is the first study to provide insight into the treatment course of patients with secondary LLL.

### Characteristics of the participants

The age at initial diagnosis and the percentage of cancer types among the participants in this study were similar to those in previous epidemiological studies examining the incidence of secondary LLL after treatment for gynecological cancer in Japan [4, 5]. Concerning the time to onset of lymphedema, it has been reported that 85.2% of patients who developed lymphedema were diagnosed within 2 years of treatment [4], and the median time from surgery to lymphedema onset was 4.2 to 6.8 months [5]. On the other hand, regarding the time from surgery to the first visit for LLL, another previous study that examined the effect of treatment for LLL reported a median of 55.3 months [14], which was similar to the results of the present study. Furthermore, nearly half (48.0%) of the patients had their first visit more than 5 years after surgery in this study, and, as in previous studies, the patients in our study were also seen after some time had elapsed postoperatively. Therefore, even if most patients developed edema at a relatively early stage, it is possible that they observed the progress of edema on their own while it was mild and visited a specialized hospital only when it became significantly noticeable.

In this study, ISL staging was used to assess the severity of lymphedema, rather than the percentage of excess volume (PEV) in the affected limb, since patients with bilateral as well as unilateral lymphedema were included in the study. The participants in this study tended to have both early stage II (60.8%) and late stage II (39.2%) lymphedema, and among stage II patients, those with relatively mild disease tended to be more common.

### Optimal model of IRLV based on GBTM analysis

The mean rate of lower extremity volume loss over time has been examined in a previous study by Kim et al. [14]. In this study, GBTM was used to examine the treatment course in greater detail. We compared our study with this previous study since the end of intensive care in the previous study [14] corresponded to the 3-month point after the intervention in the present study. Comparison showed that the trajectory of the moderate response group in this study was most similar to the results of the previous study. However, the standard deviation of the percentage reduction in affected limb volume at each assessment time point in the previous study was large. In other words, there might have been potential treatment progress patterns that could not be aggregated into the mean. The GBTM analysis in this present study newly revealed the existence of three response trajectories. We consider that this is an important finding for the development of highly individualized treatment plans that include various treatment courses. Furthermore, in addition to showing the importance of treatment adherence, our study results suggest the possibility of predicting subsequent progress at 3 months of intervention. Thus, if at 3 months post-intervention, the affected limb volume increases and the physician determines that compression therapy adherence is poor, and reteaching the patient about self-management methods might improve subsequent progress.

### Factors associated with each trajectory of IRLV

In this study, compression therapy adherence at 3 months after intervention was observed to be a predictor of the treatment effectiveness. The World Health Organization stated that adherence needs to be assessed for effective and efficient treatment [25]. Boris et al. [21], who examined the relationship between adherence and the effectiveness of complex lymphedema therapy in patients with lymphedema of the extremities, reported that patient adherence to treatment influenced the maintenance of volume reduction, with patients who were more adherent more likely to achieve edema reduction. Another study by Forner-Codero et al. [20], which examined predictors of response to CDT in patients with lymphedema of the upper extremity secondary to treatment for breast cancer, involved an inpatient intervention in which the duration of daily compression therapy was assessed by medical personnel and was analyzed at three levels based on the duration of therapy: good, partial, and poor. They reported a 25% increase in volume reduction with good adherence to compression therapy compared to poor adherence. The 2020 International Lymphatic Association Consensus Document also states that patient adherence is essential for improving treatment efficacy [1], and the results of our study also support previous research that patient adherence is an important factor related to treatment efficacy.

### Limitations and future issues

The first limitation of this study is that it was a retrospective study that was conducted at a single center. Additionally, many subjects in this study were referred from other hospitals, and there were items that could not be examined, such as body mass index and the number of lymph nodes removed. These factors have been identified as important factors related to LLL in previous studies and should be the subject of future research [26, 27]. Second, regarding the evaluation of adherence to compression therapy, a previous study conducted evaluations based on the hours of daily compression therapy implementation, whereas this study only evaluated whether or not it was implemented during the day and at night [21]. Based on the above, it is necessary to conduct evaluation interventions from the preoperative period and conduct an adherence assessment based on detailed compression therapy implementation time in the future. Finally, the sample size of this study is a potential limitation. Since the sample size required for GBTM analysis is considered as 100 or more cases [23], the sample size of this study was considered appropriate for GBTM analysis. However, we consider that further study with a larger sample size is necessary to conduct a more accurate analysis and to estimate and extract potential patterns of progress and related factors that might not have been revealed in this study.

In conclusion, GBTM analysis of the effect of outpatient-based complex decongestive therapy on postoperative LLL in patients operated on for gynecologic cancer indicated three response trajectory patterns following treatment. Moreover, adherence to compression therapy at 3 months post-intervention was extracted as a factor associated with no response to CDT.

## Data Availability

The participants of this study did not agree for their data to be shared publicly, so the data of participants are not available.
